# Giant mucocele originating from the middle concha in a 5-year-old child: a case report

**DOI:** 10.1186/1752-1947-7-246

**Published:** 2013-10-18

**Authors:** Gaffar Aslan, Mehmet Birol Ugur, Nuray Başsüllü

**Affiliations:** 1 Department of ENT, Faculty of Medicine, Bilim University, Istanbul, Turkey; 2 Department of ENT, Faculty of Medicine, Gazi University, Ankara, Turkey; 3 Department of Pathology, Faculty of Medicine, Bilim University, Istanbul, Turkey; 4 GaziÜniversitesi, TıpFakültesi, KBB AD ÖğretimÜyesi, Ankara, Turkey

**Keywords:** Child, Computed tomography, Endoscopic nasal surgery, Middle concha, Mucocele, Nasal obstruction

## Abstract

**Introduction:**

Mucoceles are mucus-filled, epithelial-lined sacs that slowly develop in the paranasal sinuses when sinus or concha bullosa drainage is obstructed by inflammatory processes, trauma, or prior surgery. They are extremely rare in children. Symptoms usually arise from the nasal obstruction or compression of neighboring structures.

**Case presentation:**

This case report describes a 5-year-old Turkish boy with a 3-year history of nasal obstruction. A computed tomography scan showed a well-defined soft tissue density lesion, seemingly originating in the region of the middle concha and was suggestive of a middle concha mucocele. The mass was removed by endoscopic sinus surgery.

**Conclusions:**

In the case of a child presenting with nasal obstruction, mucocele should be remembered in the differential diagnosis of intranasal tumors. Computed tomography and magnetic resonance imaging are helpful in making the diagnosis and endoscopic nasal surgery has proven successful in the treatment.

## Introduction

Langenback first described mucoceles in 1819, but it was Rollet who introduced the term mucocele in 1896. Onodi described the histological characteristics and Turner differentiated the frontal from the ethmoidal lesion [1]. Mucoceles are usually seen in adults and are rare in babies and children [2]. The etiologic mechanism of mucoceles is not fully understood, but obstruction of the sinus ostium due to chronic rhinosinusitis, nasal polyps, or tumors, possibly result in an accumulation of secretions and an expanding mass. Previous sinus surgery can also result in ostium obstruction and subsequent mucocele development [3]. When seen in children, they are linked with cystic fibrosis [4]. Although not fully understood, it has been hypothesized that mucosal stasis in cystic fibrosis leads to their formation [5].

## Case presentation

**Figure 1:**
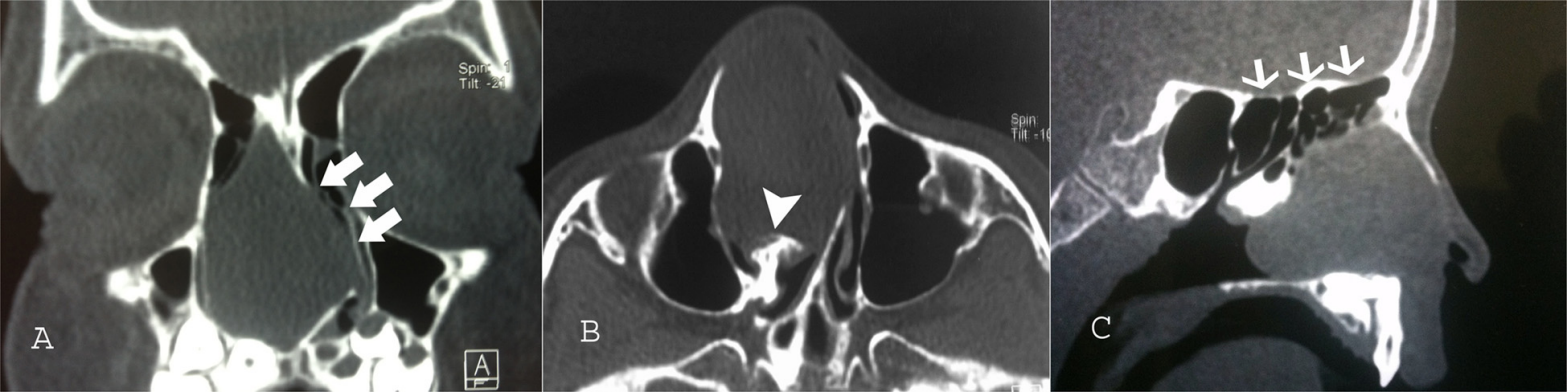
**Coronal (A), axial (B), and sagittal (B,C) paranasal sinus computed tomography images showing a mucocele of the nasal cavity. (A)** Coronal image showing the extensive mucocele filling the nasal cavity without orbital extension. The nasal septum has deviated to the left side due to the mass effect (white arrows). **(B)** Axial image showing the originating site of the mucocele from the middle concha (white arrowhead). **(C)** Sagittal image of the mucocele. Note that the cranial base is intact without extension into the cranial fossa (thin white arrows).

**Figure 2:**
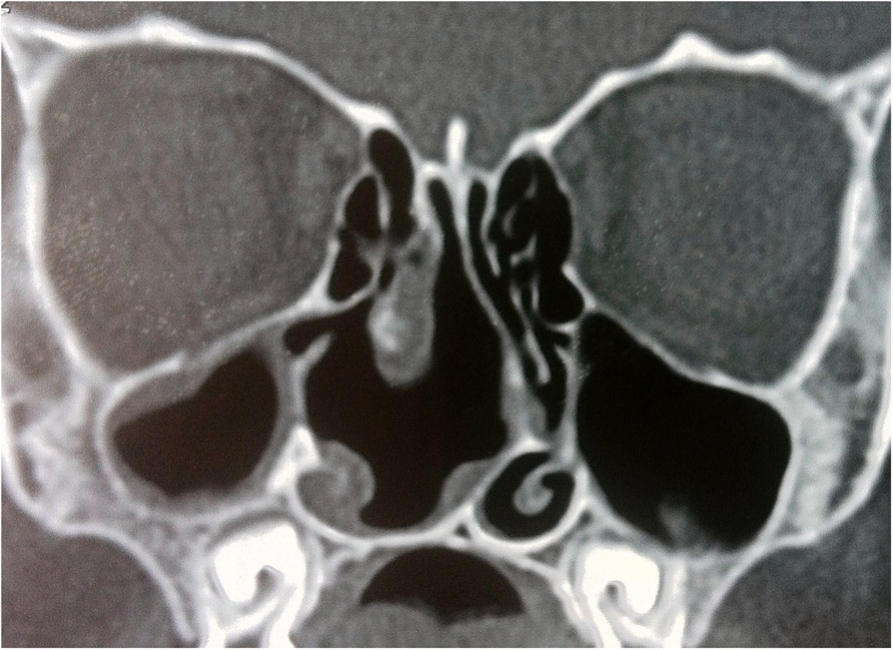
Coronal paranasal sinus computed tomography images 2 months after the operation showing no signs of recurrence or inflammation.

**Figure 3:**
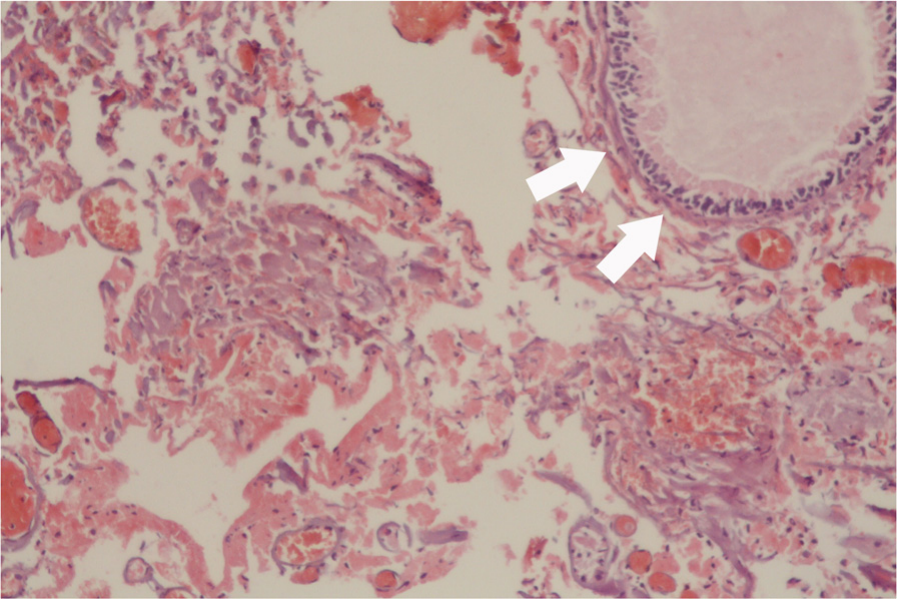
**Histopathologic microphotograph of the benign cyst wall.** The microphotograph shows a benign cyst lined by ciliated columnar mucin-secreting cells (white arrows) with no secondary changes due to infection or hemorrhage; dyed with hematoxylin and eosin stain under 40 × magnification.

A 5-year-old Turkish boy presented with a 3-year history of nasal obstruction. He was otherwise healthy and had no fever, recent cough, nasal discharge, or epistaxis. On physical examination, a pink soft tissue mass in his right nostril was visible. His nasal septum was completely deviated to the left side and there was slight nasal enlargement due to the mass. A needle aspiration from the mass revealed a clear fluid with shrinkage of the mass, indicating that the mass was a cystic lesion rather than a soft tissue one. Other head and neck region examination findings were normal. His white blood cell count was 11.5×10^3^/mm^3^ (range: 4 to 10×10^3^/mm^3^), hemoglobin level 13.4g/dL (11 to 16g/dL), platelets 361×10^9^/L (range: 150 to 400×10^9^/L), and sedimentation rate 9 (range: 3 to 20mm/hour). A high resolution computed tomography (HRCT) scan showed a well-defined soft tissue density lesion, seemingly originating in the region of the middle concha and was suggestive of a middle concha mucocele (Figure 1A and 1B). Sagittal plane images excluded any intracranial extension (Figure 1C). Our plan was to remove the mass by endoscopic sinus surgery. Intraoperatively, we found an extensive cyst filling the right nasal cavity, originating from the middle concha. The anteroinferior and mediolateral walls of the cyst were removed, effectively marsupializing it into the nasal cavity. There was no erosion on the lateral nasal wall, ethmoid roof, or septum. His right nasal cavity was packed with NASOPORE^®^ (a biodegradable/fragmentable, synthetic polyurethane foam; Polyganics, Groningen, The Netherlands), which was aspirated on the tenth postoperative day. The postoperative period was uneventful without any complications or synechia formation. His nasal obstruction improved remarkably in the immediate postoperative period. A postoperative HRCT examination was performed 2 months after surgery. There were no signs of recurrence or inflammation (Figure 2). Histopathology revealed a benign cyst lined by ciliated columnar mucin-secreting cells with no secondary changes due to infection or hemorrhage (Figure 3). On follow-up, he was disease free at the end of 18 months.

## Discussion

Mucoceles are mucus-filled, epithelial-lined sacs that occur in paranasal sinuses. They are benign lesions that gradually expand via bony reabsorption and erosion with resultant new bone formation [4].

Mucoceles are unilateral in 90% of cases. Between 60% and 65% of mucoceles reside in the frontal sinus, 20% to 25% in the ethmoidal sinus, 5% to 10% in the maxillary sinus and 5% to 10% in the sphenoidal sinus. In adults, the most common location is the frontoethmoidal region, followed by the maxillary sinus, sphenoid sinus, and posterior ethmoid [4,6]. Nicollas *et al*. reported a series of 10 cases of paranasal sinus mucoceles in children. Cystic fibrosis was found in six cases out of 10. The mucoceles had originated from the ethmoid sinus in eight of the 10 cases in this series [4].

Mucoceles progress slowly and their associated clinical symptoms depend on their location and extent of expansion. Some patients complain only of nasal obstruction and persistent nonspecific headache, whereas others have painless orbital swelling, diplopia, visual field disturbance, or globe displacement [2,7-10]. Sinus ostium obstruction due to chronic rhinosinusitis, nasal polyps, or tumors, may cause the accumulation of secretions and an expanding mass. Previous sinus surgery can also result in ostium obstruction and subsequent mucocele development [3]. Our patient presented with slight nasal enlargement and nasal obstruction and had no history of trauma or previous surgery.

An HRCT scan is the preferred diagnostic imaging modality. On HRCT, mucoceles appear as a homogenous round or oval mass with surrounding bony erosion. The differentiation between a mucocele versus an expansile mass may be made with magnetic resonance imaging (MRI). MRI is helpful in clarifying soft tissue extension to the cranium and orbit. It also helps in differentiating from other soft tissue masses with potentially similar presentation such as meningocele, rhabdomyosarcoma, hemangioma, and neuroblastoma [2,10,11]. In our patient we did not perform MRI, as CT imaging had already excluded the possibility of intracranial and orbital extension. Because the needle aspiration ensured the lesion was a cyst we did not need to differentiate it from other soft tissue masses.

All cases of mucocele should be treated surgically. Two methods may be used in the treatment of paranasal sinus mucocele. One of them, conservative minimally invasive surgery, involves endoscopic marsupialization of the mucocele. This approach preserves the nasal anatomy and architecture. The other approach is the more radical transfacial approach and involves a Lynch–Howarth incision that is used mostly in cases with intracranial extension [12,13]. In our case, we chose to perform an endoscopic management as a more conservative approach.

The cyst is mainly lined by pseudostratified ciliated columnar or cuboidal epithelium, but sometimes it may be lined with areas of squamous epithelium. There is cellular infiltrate comprising neutrophils, lymphocytes, plasma cells, and eosinophils. Subepithelial lymphoid aggregates and vascularity are also seen. Osseous elements are woven bone, lamellar bone, osteoblasts, and osteoclasts [14]. Our histopathology report showed a cyst-wall-like structure lined on one surface by hyalinized collagen and on the other by stratified cuboidal epithelium and the cyst involved hypocellular inflammatory cells.

## Conclusions

Mucoceles are rare in the pediatric age group. A mucocele may become large enough to make a unilateral or bilateral nasal obstruction. Therefore, in the case of a child presenting with nasal obstruction, mucocele should be remembered in the differential diagnosis of intranasal tumors. HRCT and MRI are helpful in making the diagnosis and endoscopic nasal surgery has proven successful in the treatment.

## Consent

Written informed consent was obtained from the parents of our patient for publication of this case report and any accompanying images. A copy of the written consent is available for review by the Editor-in-Chief of this journal.

## Competing interests

The authors declare that they have no competing interests.

## Authors’ contributions

GA performed the surgical procedure and obtained the patient data and was a major contributor in writing the manuscript. MBU was a major contributor in writing the manuscript. NB performed the pathologic examination and was a contributor in writing the manuscript. All authors read and approved the final manuscript.

## References

[B1] Evans C Aetiology and treatment of fronto-ethmoidal mucocele J Laryngol Otol 1981 95 361 375 10.1017/S0022215100090836 7229519

[B2] Diaz MC Schmidt RJ Ethmoid mucocele presenting as an orbital mass Pediatr Emerg Care 2008 24 845 846 10.1097/PEC.0b013e31818ea0bd 19092564

[B3] Loehrl TA Leopold DA Sphenoethmoidal mucocele presenting with bilateral visual compromise Ann Otol Rhinol Laryngol 2000 109 608 610 10855576 10.1177/000348940010900615

[B4] Nicollas R Facon F Sudre-Levillain I Forman C Roman S Triglia JM Pediatric paranasal sinus mucoceles: etiologic factors, management and outcome Int J Pediatr Otorhinolaryngol 2006 70 905 908 10.1016/j.ijporl.2005.10.002 16293319

[B5] Alvarez RJ Liu NJ Isaacson G Pediatric ethmoid mucoceles in cystic fibrosis: long-term follow-up of reported cases Ear Nose Throat J 1997 76 538 539 543–536 9282461

[B6] Hartley BE Lund VJ Endoscopic drainage of pediatric paranasal sinus mucoceles Int J Pediatr Otorhinolaryngol 1999 50 109 111 10.1016/S0165-5876(99)00220-7 10576610

[B7] Haloi AK Ditchfield M Maixner W Mucocele of the sphenoid sinus Pediatr Radiol 2006 36 987 990 10.1007/s00247-006-0243-x 16802142

[B8] Moriyama H Nakajima T Honda Y Studies on mucocoeles of the ethmoid and sphenoid sinuses: analysis of 47 cases J Laryngol Otol 1992 106 23 27 10.1017/S002221510011850X 1541884

[B9] Delfini R Missori P Iannetti G Ciappetta P Cantore G Mucoceles of the paranasal sinuses with intracranial and intraorbital extension: report of 28 cases Neurosurgery 1993 32 901 906 discussion 906 10.1227/00006123-199306000-00003 8327090

[B10] Olze H Matthias C Degenhardt P Paediatric paranasal sinus mucoceles Eur J Pediatr Surg 2006 16 192 196 10.1055/s-2006-924000 16909359

[B11] Thome DC Voegels RL de la Cortina RA C Butugan O Bilateral ethmoidal mucocele in cystic fibrosis: report of a case Int J Pediatr Otorhinolaryngol 2000 55 143 148 10.1016/S0165-5876(00)00375-X 11006454

[B12] Zrada SE Isaacson GC Endoscopic treatment of pediatric ethmoid mucoceles Am J Otolaryngol 1996 17 197 201 10.1016/S0196-0709(96)90060-5 8827280

[B13] Har-El G Endoscopic management of 108 sinus mucoceles Laryngoscope 2001 111 2131 2134 10.1097/00005537-200112000-00009 11802010

[B14] Lund VJ Milroy CM Fronto-ethmoidal mucocoeles: a histopathological analysis J Laryngol Otol 1991 105 921 923 10.1017/S0022215100117827 1761945

